# Carriage of Antibiotic‐Resistant *E*. *Coli* by Mixed Dog Breeds in Kasarani Subcounty, Kenya: A Public Health Concern

**DOI:** 10.1155/vmi/5305098

**Published:** 2026-04-03

**Authors:** Ombura Esther Bosibori, Lilly Carolyne Bebora, Susan Mbugua

**Affiliations:** ^1^ Department of Veterinary Public Health, Pharmacology and Toxicology, University of Nairobi, P.O. Box 29053-00625 Kangemi, Nairobi, Kenya, uonbi.ac.ke; ^2^ Department of Veterinary Pathology, Microbiology and Parasitology, University of Nairobi, P.O. Box 29053-00625 Kangemi, Nairobi, Kenya, uonbi.ac.ke; ^3^ Department of Veterinary Clinical Studies, University of Nairobi, P.O. Box 29053-00625 Kangemi, Nairobi, Kenya, uonbi.ac.ke

**Keywords:** antibiotic resistance, dogs, *E. coli*, Kasarani

## Abstract

Antibiotic resistance (AMR) is currently one of the most worrying trends globally, as it prevents effective treatment of diseases, some of which may be life‐threatening. Pet animals are assumed to be potential reservoirs for transferring AMR to humans due to the wide use of antibiotics on pets and their close contact with humans. This study confirmed the carriage of *E. coli*‐resistant bacteria by dogs in the Kasarani area, Nairobi, Kenya. Fecal samples were collected from 18 (11 stray dogs and 7 home‐kept) dogs. The samples (14) yielded *E. coli*, which were further tested for susceptibility to selected antibiotics, by the agar disc diffusion method; 13 of them demonstrated resistance at various rates: highest resistance was to ampicillin (AMP) at 85.7%, followed by sulfamethoxazole (SX) at 64.3%, cotrimoxazole (COT) and tetracycline (TE) at 57.1%, streptomycin (S) at 50%, kanamycin at 21.4%, and chloramphenicol at 7.1% (1 isolate); while all isolates were susceptible to gentamycin, 3 isolates were resistant to AMP only, one of which was resistant to 6 antibiotics, while one was susceptible to all 8 antibiotics tested. Some isolates demonstrated multiple resistance; one showed resistance to 6 antibiotics tested. Antibiotics with high inclusion in the multiresistant strains were AMP and TE at 72.7% (8/11) each. The next common inclusion was SX at 63.6% (7/11), followed by COT and S at 54.5% (6/11) each. The bacteria resistant to chloramphenicol were further resistant to four other antibiotics: AMP, COT, S, and SX. Results of this study could help guide the empirical use of antibiotics in small animal practice and further provide added information on the status of AMR bacteria in Kasarani.

## 1. Introduction

Antibiotic resistance (AMR) is currently a big threat to global health. It has risen to dangerously high levels globally, making it difficult to treat infectious diseases [1–3]. AMR has resulted in patients incurring extra expenses as they have to buy more expensive medicines, mainly reserved for life‐threatening infections that are difficult to treat. The patients also stay longer in hospitals due to ineffective medicines, thus translating to higher hospital bills [4, 5]. Many public health organizations have described the rapid emergence of resistant bacteria as a “crisis” that could have “catastrophic consequences” [6]. In 2013, the Centers for Disease Control and Prevention [7] declared that the human race is now in the “postantibiotic era,” and in 2014, the World Health Organization (WHO) warned that the AMR crisis is becoming dire. The high rate of AMR development in bacteria has alerted World bodies such as the WHO, Food and Agriculture Organization (FAO) of the United States, and the World Organization of Animal Health (WOAH), who have now found it necessary to unite and combat it as a joint force [2, 3, 8, 9]. The current estimation is that, globally, 700,000 patients die annually due to resistant infections; if nothing is done to combat the development of AMR in bacteria, the death rate could easily escalate to 10 million annually by the year 2050 [10].

Antibiotics are also used in animals to treat animal diseases and as growth promoters, mostly in chickens and pigs, to increase productivity [11]. One health concept came up when it was noted that animals share the most pathogenic bacteria with humans, which account for about 60% of human bacterial pathogens [12]. Since the same antibiotics are used to treat diseases in humans and animals [1, 12], once resistance develops in animal bacteria, it can easily be passed on to humans, and the vice versa is also possible [13, 14]. One animal that may worsen the situation by criss‐crossing between humans and animals is the dog [15].

Dogs are found everywhere, and they have long been considered “man’s best friend,” for companionship and as guard dogs [15]. The closeness between dogs and humans makes it easy for cross‐transfers of bacteria and other pathogens between them. These dogs roam around in villages or peri‐urban areas; thus, if they carry AMR bacteria, they disseminate them as they defecate. The situation is similar for dogs confined in homes, as they are walked out along roads and streets where they defecate, thus contributing to the possible dissemination of resistant bacteria. Farmers, including nomadic ones, also use dogs to look after and protect their livestock, facilitating the cross‐transfer of bacteria. It is also easy for dogs to pick up antibiotics and/or resistant bacteria from a contaminated environment ([16]; [17]; [18]). The contamination could be from human fecal material or drugs carelessly disposed of.

Pet animals have been documented as reservoirs of AMR bacteria [19–21], while there is scant information on AMR in stray dogs. This is because more attention has been given to pet welfare, due to the substantial increase in pet animal numbers in modern society. Antibiotics are frequently used in small animal veterinary practice [11], often including those that are used in human medicine, with heavy usage of broad‐spectrum ones such as aminopenicillins plus clavulanic acid, cephalosporins, and fluoroquinolones.

Therefore, this study focused on the dog to see if it carries AMR bacteria, taking *Escherichia coli* (*E. coli*) as the indicator bacteria. Most antibiotic susceptibility studies are performed using *E. coli* because they are the most prevalent enteric bacteria in both animals and humans and are also important zoonotic agents that can be implicated in both animal and human infections [22]. The organisms can also be taken as a good indicator of the potential presence of disease caused by bacteria and show the general sanitary quality of food since they are closely associated with fecal contamination [22]; they are also easy to grow. Therefore*, E. coli* would efficiently serve as a representative for the other bacteria within the same individual/environment, assuming that, if resistance genes are present, the bacteria can easily transfer them to other bacteria, including the pathogenic ones [13, 23].

## 2. Materials and Methods

### 2.1. Study Area

**FIGURE 1:**
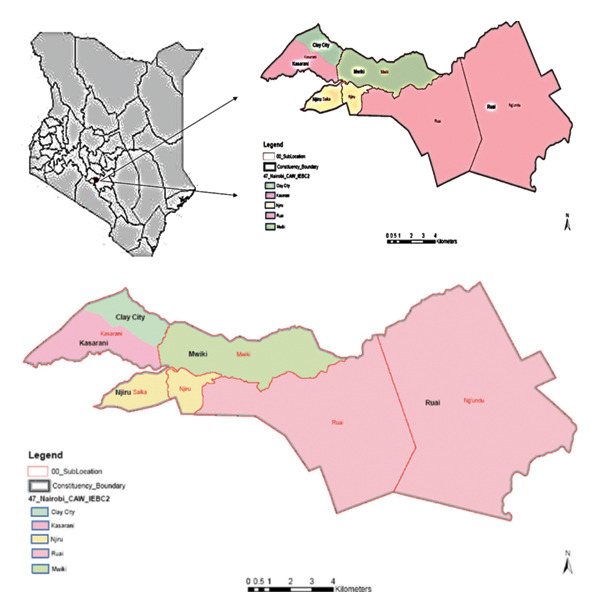
Map of Kasarani constituency, Kenya.

Kasarani (Figure 1) is located along Thika road, approximately 16 km by road, northeast of Nairobi’s Central Business District. The area has a population of about 260,000 as per the 2019 census data. It is at latitude 1°13′40.23’’S and longitude 36°54′20.62’’E. The constituency is divided into various wards, which include the following: Kasarani, Clay City, Mwiki, Njiru, and Ruai. All classes of people live in this area; there are high‐, middle‐ and low‐income earners in the area.

There is no specific approximate number of dogs in the Kasarani area, but there are a large number of animals, including cattle (4828), goats (4063), sheep (2672), poultry (136725), pigs (3661), donkeys (47), and cats and dogs in the area [24]. Some of the dogs are strictly home kept, some are stray, and others are home kept but keep wandering in the streets and dump sites in search of food and water. These wandering dogs always interact with humans near hotels, at the garage, marketplaces, dumping sites, and homes. Some owners keep them as pets, but the majority keep them for security purposes.

### 2.2. Sample Collection and Handling

Ethical clearance for the use of animals in this study was sought and approved by the Biosafety, Animal Use and Ethics Committee of the Faculty of Veterinary Medicine, University of Nairobi, under REF: FVMBAUEC/2023/449. The sampling was initially pegged at 30 samples from stray dogs (those that were easy to handle), and home‐kept dogs, whose owners were willing to have them tested (convenience study), but could only manage to sample 18 dogs, including pure local breeds, mixed local breeds, mixed Japanese spitz breed, and German shepherds, due to convenience and willingness.

Eighteen fecal samples were aseptically collected (December 2017) from both stray (11) and home kept (7) dogs, males and females, put in plastic fecal bottles, and immediately transported in a cool box to the laboratory at the Department of Veterinary Pathology, Microbiology and Parasitology, University of Nairobi, Kabete, for isolation and identification of *E. coli* bacteria. Samples that were not processed immediately were refrigerated at 4°C.

### 2.3. Isolation and Characterization of *E. coli*

Isolation was performed as prescribed by Bergey’s Manual of Determinative Bacteriology [25]. Using MacConkey agar (Oxoid, Basingstoke, United Kingdom), the sample was inoculated and incubated overnight at 37°C. Lactose fermenting (pink color), medium‐sized colonies were further characterized as *E. coli* using standard biochemical tests. *E. coli* are Gram‐negative rods, motile, oxidase‐negative, catalase‐positive, gelatinase‐negative, urease‐negative, indole‐positive, methyl red−positive, Voges−Proskauer test−negative, and citrate utilization−negative. All *E. coli* isolates were tested for susceptibility to selected antibiotics; no attempt was made to check whether they were pathogenic strains or commensals.

### 2.4. Antibiotic Susceptibility Testing (AST)

AST was performed by the agar disk diffusion method as previously described by [26] and recommended by the Clinical and Laboratory Standards Institute [27]. Bacterial suspensions were inoculated onto Mueller−Hinton agar (MHA) (Oxoid, Basingstoke, United Kingdom) plates by the streaking method using cotton swabs. In brief, after dipping into the bacterial suspension 1.5 × 10^8^ colony‐forming units (CFUs)/mL (turbidity equivalent to 0.5 MacFarland standard), the swab was firmly pressed against the side of the tube to remove excess inoculum. The swab was then repeatedly streaked on the MHA medium, rotating the plate approximately 60° each time to ensure even distribution of the inoculum, for confluent growth.

The *E. coli* isolates were tested for susceptibility against 8 antibiotics, commonly used in humans and animals, including ampicillin (AMP) (25 mcg), tetracycline (TE) (25 mcg), cotrimoxazole (COT) (25 mcg), streptomycin (S) (10 mcg), kanamycin (30 mcg), gentamycin (10 mcg), sulfamethoxazole (SX) (200 mcg), and chloramphenicol (30 mcg). Respective antibiotic discs, manufactured by HiMedia Laboratories, India, were used for this testing. After streaking, the antibiotic discs were placed on the agar using sterile forceps; the agar plate was then incubated aerobically at 37°C overnight. After incubation, the diameters of the growth‐inhibition zones around the discs were measured using a Vernier caliper. A standard *E*. *coli* (*E. coli*‐ATCC 25922 [8]) was used as the reference strain. Guidelines provided by the CLSI [27] were used to interpret the inhibition zone results, the size of the inhibition zone being directly proportional to the susceptibility of the organism to the particular antibiotic [28]. However, in this study, by design, isolates resistant to two or more antibiotics were considered to be multidrug‐resistant [29].

NB: Classes of antibiotics as used in the study. AMP‐ beta‐lactam;TE‐tetracyclines; COT and SX‐sulfonamides; chloramphenicol‐amphenicol; streptomycin, kanamycin and gentamycin‐aminoglycosides.

## 3. Results

### 3.1. *E. coli* Isolates

Of the 18 fecal samples cultured, 14 yielded *E. coli*. This translates to 77.8% recovery.

### 3.2. AST Results

**FIGURE 2:**
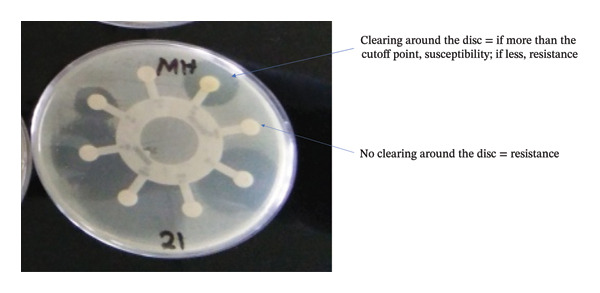
Antibiotic susceptibility/resistance results of one *E. coli* isolate.

AST results of the 14 *E. coli* isolates are given in Table 1 [27]. The organisms showed the highest resistance to AMP at 85.7%, followed by SX at 64.3%, COT and TE at 57.1% each, and S at 50%. Low resistance was demonstrated to kanamycin at 21.4% and chloramphenicol at 7.1% (1 isolate), while all isolates were susceptible to gentamycin. The 3 isolates were resistant to AMP only; one was resistant to 6 antibiotics, while one was susceptible to all the 8 antibiotics tested. Figure 2 shows the antibiotic susceptibility results of one of the isolates.

**TABLE 1:** Antibiotic resistance patterns of the isolated *E. coli*: *n* = 14.

Antibiotic	Susceptible		Resistant	
	Number	Percent	Number	Percent
Ampicillin	2	14.3	12	85.7
Tetracycline	6	42.9	8	57.1
Cotrimoxazole	6	42.9	8	57.1
Streptomycin	7	50	7	50
Kanamycin	11	78.6	3	21.4
Gentamycin	14	100	0	0
Sulfamethoxazole	5	35.7	9	64.3
Chloramphenicol	13	92.9	1	7.1

### 3.3. Multidrug Resistance (MDR)

Eleven of the isolates demonstrated multiple resistance (i.e., resistance to two or more antibiotics), as shown in Table 2: one sample (7.1%) showed resistance to 2 antibiotics, 3 samples (21.4%) showed resistance to 4 antibiotics, 5 samples (35.7%) showed resistance to 5 antibiotics, while 1 sample (7.1%) showed resistance to 6 of the antibiotics tested. Antibiotics with the highest inclusion in the multiresistant strains were AMP and TE at 72.7% (8/11) each. The next common inclusion was SX at 63.6% (7/11), followed by COT and S at 54.5% (6/11) each. The one resistant to chloramphenicol was resistant to four other antibiotics: AMP, COT, S, and SX.

**TABLE 2:** Multidrug resistance patterns demonstrated by the isolates: *n* = 14.

Number of antibiotics resistant to *E*.*coli*	Number of isolates showing resistance (%)	Multiresistance pattern
2	1 (7.1)	Resistant to COT and SX
4	3 (21.4)	One had a combination of AMP‐TE‐S‐K Two had a combination of AMP‐TE‐COT‐SX
5	5 (35.7)	3 had a combination of AMP‐TE‐COT‐S‐SX One had a combination of AMP‐COT‐S‐SX‐C One had a combination of AMP‐TE‐S‐K‐SX
6	1 (7.1)	Resistant to AMP‐TE‐COT‐S‐K‐SX

*Note:* C = chloramphenicol; S = streptomycin; K = kanamycin; TE = tetracycline; COT = cotrimoxazole; SX = sulfamethoxazole; AMP = ampicillin.

## 4. Discussion

Results of this study showed that *E. coli* isolates from the screened dogs were resistant, though at varying levels, to some of the tested antibiotics; more so, the affordable ones, hence, comfortably accessible to the inhabitants of the study area. The resistance may have developed due to high or indiscriminate usage of antibiotics in the region, either on dogs, humans, or other animals in or traversing the region. The dogs/humans/other animals may excrete the resistant organisms and contaminate the environment where the resident dogs feed.

Many classes of antibiotics have been used to treat both humans and livestock [30]; they include the following: β‐lactams (penicillins and cephalosporins), sulfonamides with and without trimethoprim, TEs, macrolides, lincosamides and streptogramins, and quinolones including fluoroquinolones [26], [31]. Plant extracts have also successfully treated several bacterial infections [32]. Antibiotic classes most used to treat livestock are as follows: penicillin derivatives, such as AMP and cloxacillin, and sulfonamide, e.g., tylosin, used for the treatment of metritis and acute mastitis in cattle, sheep, and goats; enteritis; pneumonia; erysipelas; and infectious arthritis in swine [33]. Tylosin is also used to treat chronic respiratory disease in chickens(49). TE and COT (containing SX and trimethoprim) are the two most‐used antibiotics for prophylaxis and as growth promoters in livestock rearing. They are also used to treat infections related to acquired immunodeficiency syndrome (AIDS) in humans [34]. Penicillin has been used to treat gonorrhea in humans; however, it has increasingly become resistant [35].

An estimated 1.6 million people live with HIV/AIDS in Kenya ([36] Data Book; National Aids Control Council Report 2018); it is rated the fourth highest in terms of global epidemic ([36] Data Book). HIV/AIDS prevalence for Nairobi County is estimated to be at 6.1%. While this is lower than that of some other counties, e.g., Busia 7.7%, Siaya 21.0%, Migori 13.5%, Kisumu 16.3%, and Homa Bay 20.7% (National Aids Control Council report 2018), the free movement across the country makes it easy for HIV‐infected individuals to move around the country and beyond. Moreover, 6.1% of a large population translates to a high figure. COT (SXT) is used for treatment and prophylaxis of HIV‐associated opportunistic infections (primary or secondary) [4, 37–39]. It has been recognized for prolonging the life span of HIV‐positive individuals in resource‐limited settings such as Kenya; its affordability makes it the antibiotic of choice in countries with high prevalence of HIV. Therefore, the detection of bacteria that are resistant to it is not surprising. The isolates have shown high susceptibilities to antibiotics which are not commonly used, for example: gentamycin (100%), chloramphenicol (92.9%), and kanamycin (78.6%). The 100% susceptibility could be due to a lack of exposure to antibiotic drugs or the absence of mobile genetic elements encoding resistance. Therefore, the possibility of successful antibiotic therapy in these situations is highlighted by the indication that the isolates have maintained their wild‐type phenotype and have not been subjected to selective pressure.

Although the samples were collected from dogs, the results can be taken to reflect on the human population due to the close association of humans with dogs, for example: foods eaten, and sharing of a home or environment, which allows the transfer of the bacteria between the two populations [15].

Dogs have been repeatedly mentioned as comprising reservoirs for zoonotic and livestock‐infecting pathogens(48) and, as such, are useful for respective epidemiological monitoring. Transfer of resistant bacteria between humans and their dogs has been documented in various studies from the Western world [19]. Albrechtova et al. [41], working in Northern Kenya, an area inhabited mainly by nomadic herders, demonstrated the presence of *E. coli* strains that were resistant to various antibiotics; some of the isolates were MDR.

The researchers also specifically looked for ESBL‐producing *E. coli* strains in the area and demonstrated their presence in humans, dogs and, to a lesser extent, in cats [41]. Comparing PFGE profiles of the ESBL‐producing *E. coli* isolates, 8 isolates from dogs and 2 isolates from humans gave identical profiles; while a close relationship (> 95% relationship) was found in one human isolate and one cat isolate. This indicates the spread of multiresistant bacteria between humans and dogs.

The relatively low resistance in isolates from cats compared to the resistance rates in isolates from dogs, observed by Albrechtova et al. [41], could be explained by the different feeding habits of the two species. While cats prefer hunting for food, dogs tend to be more reliant on household leftovers; they are, therefore, more prone to coprophagy; such scavenger behavior renders dogs good sentinels of environmental contamination, including the presence of AMR bacteria.

MDR was also recorded in *E. coli* in this study to 2, 4, 5, and 6 antibiotics (Table 2). This is for the number of antibiotics tested; there could have been more if more antibiotics were tested. Considering the multiple AMR combinations found, antibiotics with high inclusion in the MDR strains were AMP and TE at 72.7% (8/11) each. The next common inclusion was SX at 63.6% (7/11), followed by COT and S at 54.5% (6/11) each. The one that was resistant to chloramphenicol was resistant to four other antibiotics: AMP, COT, S, and SX. This further demonstrates the resistance pattern being toward cheap commonly used antibiotics, echoing the global worry toward AMR [1–3]. Chances are that all the resistant traits, in a bacterium, are on one plasmid, hence transferred as a block, as is the case of methicillin resistance in *Staphylococcus* spp. [42–44]).

## 5. Conclusion

Although the sample size was small, it is believed that the results give a picture of antibiotic activities/exposures in the study area; all of the isolates were resistant to at least one antibiotic. This confirms that dogs in Kasarani are carriers of AMR bacteria; they may, thus, be part of the system that is distributing the organisms in the environment and end up infecting the human residents. Worse still is the fact that the study dogs were found to carry *E. coli* organisms, which were multiresistant to up to 6 antibiotics, noting that the number could have been even more if more antibiotics were tested. These resistant bacteria, which may be occurring as commensals in the dogs, can easily transfer the resistance factor vertically and/or horizontally between various bacterial species and genera [13, 23, 45, 46], especially to pathogenic organisms of the dogs and humans. Results of this study could help guide the empirical use of antibiotics in small animal practice and further provide added information on the status of AMR bacteria in Kasarani, and thus help inform policymakers as they embark on their fight toward AMR reduction.

## Author Contributions

Ombura Esther Bosibori, Lilly Carolyne Bebora, and Susan Mbugua contributed to conceptualization and methodology. Ombura Esther Bosibori, Mr Weda, Mrs Ann, and George Ndimbo (laboratory technicians) were involved in the investigation and laboratory work. Ombura Esther Bosibori, Lilly Carolyne Bebora, and Susan Mbugua were involved in writing of the original draft and presentation. Lilly Carolyne Bebora and Susan Mbugua supervised the study.

## Funding

No funding was received for this study.

## Disclosure

The authors would also like to acknowledge the presence of “Antibiotic resistance profiles of *E.coli* isolated from pooled samples of sick, farm, and market chicken in Nairobi County” as a preprint at the Research Square, which has similarities with our study.

## Conflicts of Interest

The authors declare no conflicts of interest.

## Data Availability

Any additional data are available upon request via the corresponding author.
